# Oral Fine Motor Control in Patients With Anterior Maxillary Single Implants, Freestanding Teeth and Teeth Connected in 3‐Unit Bridges: An Experimental In Vivo Study

**DOI:** 10.1111/clr.70136

**Published:** 2026-05-08

**Authors:** Nicole Winitsky, Aron Naimi‐Akbar, Jan‐Ivan Smedberg, Anastasios Grigoriadis

**Affiliations:** ^1^ Department of Prosthetic Dentistry Public Dental Services, Folktandvården Eastmaninstitutet Stockholm Sweden; ^2^ Department of Dental Medicine Karolinska Institutet Huddinge Sweden; ^3^ Private Practice Stockholm Sweden; ^4^ Health Technology Assessment‐Odontology (HTA‐O) Malmö University Malmö Sweden

**Keywords:** fixed partial denture, oral motor skills, single implant

## Abstract

**Objectives:**

The oral fine motor control is compromised in patients with full‐arch, bimaxillary bridges compared to those with natural teeth. No previous studies have examined the differences between the prosthetic treatments small anterior bridges and single anterior implants (SI). The aim of the study was to evaluate oral fine motor control in patients with single anterior tooth loss, comparing anterior teeth connected in 3‐unit resin bonded bridges (RBB) and free‐standing teeth adjacent to single implants (SI).

**Materials and Methods:**

A standardized hold‐and‐split task was performed in 16 patients on two occasions: once, with a RBB in place and once after replacement with a SI. The four conditions connected tooth (CT), pontic (P), free‐standing tooth (T), and single implant (SI) were tested. Outcome variables hold force, variability of hold force, split force, and duration of split were analyzed using paired sample *t*‐test or Wilcoxon signed rank test, with a significance level set at *p* < 0.05.

**Results:**

The results showed significant differences between T and SI for all variables (*p* < 0.05) except for duration of split (*p* > 0.05), and between CT and P regarding hold force and duration of split. No significant differences were found between CT and T or P and SI for any of the outcome variables (*p* > 0.05).

**Conclusions:**

Oral fine motor control on a SI was compromised compared to a T and comparable to a P of a 3‐unit RBB. However, splinting a tooth (CT) in a 3‐unit anterior RBB does not seem to compromise the oral fine motor control.

## 1 Introduction

Periodontal mechanoreceptors (PMRs) are a specialized group of receptors situated in the periodontal ligament space surrounding the roots of the teeth (Cash and Linden 1982). These receptors have an important task of transferring sensory information about the magnitude and direction of forces applied to the tooth during the initial tooth‐food contact, which is the first step in mastication (Trulsson 2005, 2006; Trulsson and Johansson 1996a, 1996b, 2002). The central nervous system uses the sensory information from the teeth to modulate and “fine‐tune” the masticatory movements (Lund et al. 1998; Lavigne et al. 1987; Lund 2011). Thus, the sensory information from the PMRs is integral for sensorimotor regulation of the biting and chewing behavior.

**FIGURE 1:**
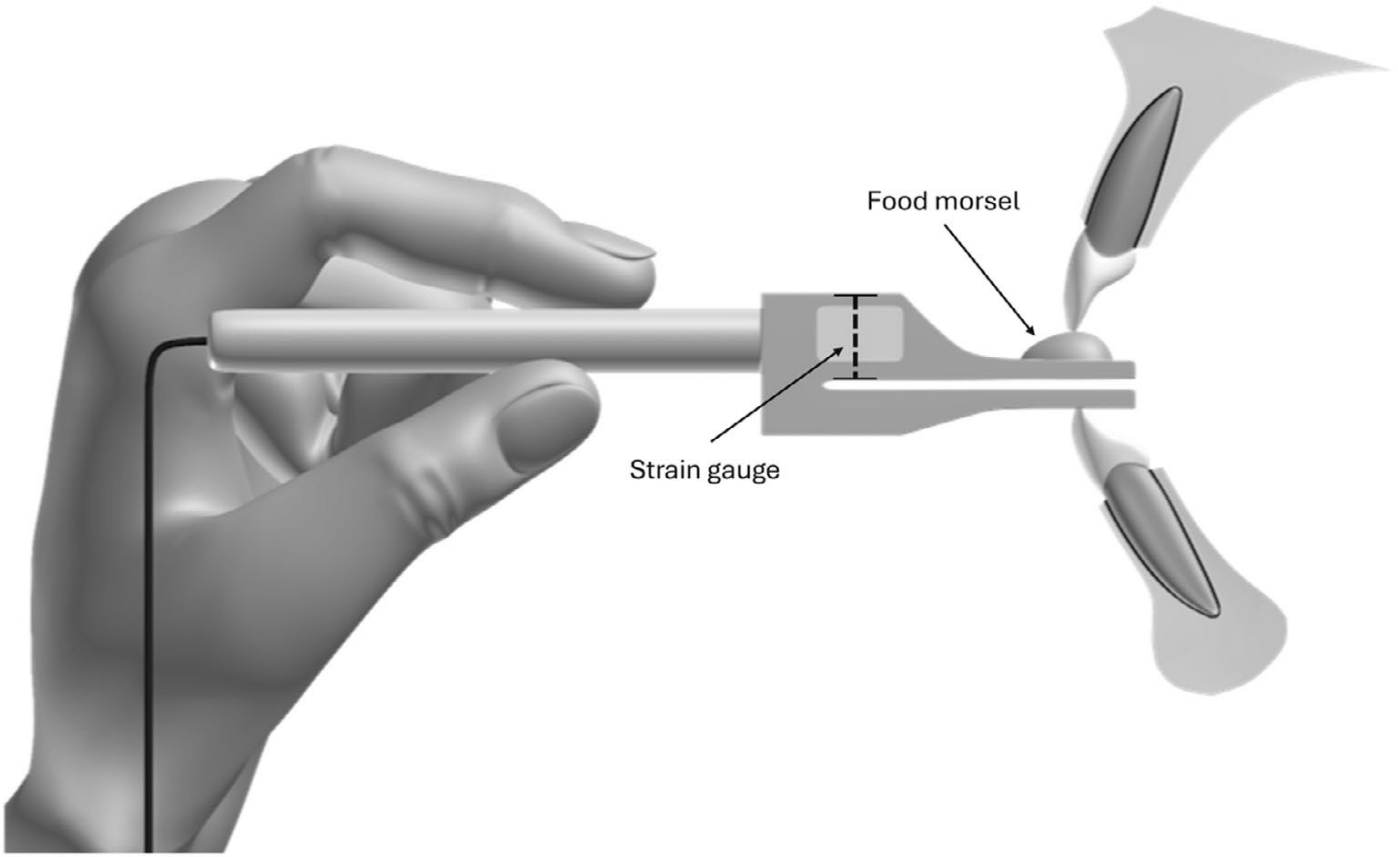
A schematic illustration of the custom‐built hold‐and‐split apparatus. The apparatus features a bar‐shaped, plastic covered metal handle, measuring 11 cm in length and 0.7 cm in diameter. Attached to the handle are duralumin blocks that terminate in two parallel rectangular plates, with a stiffness of 50 N/mm between them. The upper duralumin block incorporates a strain gauge force transducer, which alters its resistance upon compression. The resulting change in resistance is converted into an electrical signal, enabling the measurement and recording of the hold and split forces applied to the plates. The design of the apparatus is constructed to maintain consistent force measurements, regardless of the location on the plate where the force is applied.

Clinical evidence supports the involvement of periodontal mechanoreceptors (PMRs) in controlling jaw movements in people with natural dentition and dental prosthetic patients (Trulsson 2006; Trulsson et al. 2012). The role of periodontal mechanoreceptors (PMRs) in oral motor control was clinically assessed using a custom‐built “hold‐and‐split” apparatus that measured the forces involved in holding and splitting half a peanut (Figure 1) (Kumar et al. 2019; Al‐Manei et al. 2021). The “hold‐and‐split” model combines manipulation and bite force of the human biting behavior (Trulsson and Johansson 1996a). Previous studies have shown impaired force control and impaired spatial regulation due to the absence (dental implants) or alteration (dental anesthesia, periodontitis, connected teeth) of sensory inputs from the PMRs (Trulsson and Johansson 1996a; Svensson and Trulsson 2009, 2011; Grigoriadis et al. 2016; Johansson et al. 2006). Specifically, studies performed in patients with full‐arch, bimaxillary dental implant bridges, show a less exact splitting of a food morsel and an increase in the magnitude of forces applied during initial tooth‐food contact and splitting in comparison to people with natural dentition (Grigoriadis et al. 2016; Svensson and Trulsson 2011; Svensson et al. 2013). The differences in precision, as well as higher hold‐ and bite forces between patients with dental implants and people with natural dentition have been attributed to the lack of sensory inputs from the PMRs in the patients with implants. Interestingly, patients with bimaxillary tooth‐supported bridges also show signs of impaired force control. Hence, it has been suggested that splinting prosthetic units together compromise the rich sensory information provided by the PMRs and may be avoided for normal function of the masticatory system (Svensson and Trulsson 2011; Svensson et al. 2013; Svensson 2010).

In the current study, the sensorimotor regulation properties of teeth and prosthetic replacements involved in the two treatment paradigms, anterior resin‐bonded bridges (RBB) and anterior single dental implants (SI), were evaluated. The aim of the study was to compare the oral fine motor control of teeth and prosthetic replacements involved in patients with single anterior tooth loss treated with RBB in relation to SI. It was hypothesized that SIs, the pontics (of the RBBs) (P), and the teeth connected with RBBs (CT) would show impaired force control in comparison to the free‐standing teeth adjacent to the dental implants.

## 2 Materials and Methods

The study was approved from the regional ethical committee (EPN) in Stockholm (Dnr 2018/1677‐31/1). In accordance with the Helsinki declaration, all participants signed a document for informed consent, and the study was registered at the ISRCTN registry (24104637). This was an experimental in vivo study in which each participant served as their own control and was tested before and after implant placement. The inclusion criteria were patients missing one maxillary central incisor, planned for SI treatment wearing an intermediate three‐unit, metal ceramic, RBB attached to one tooth on each side of the missing tooth. Exclusion criteria were temporomandibular pathologies, subjective symptoms or malfunction when chewing or biting, allergy to peanuts, and missing or prosthetically treated occluding teeth in the anterior mandible. The patients were tested on two occasions: the first before implant treatment with the RBB in place and the second after implant treatment. This study is reported in accordance with the STROBE guidelines for cohort studies.

### 2.1 Patients

The study group included 12 males and 4 females with a mean age of 24 ± 9 (range 20–59) years, missing one anterior central incisor due to trauma. Patients were retrieved from a list of patients planned for single anterior implant treatment at two departments of Prosthetic Dentistry in Sweden between November 2020 and January 2022. A metal retention cord was bonded to the lower anterior teeth in two (12%) patients. In 10 (63%) of the patients the RBB had never been recemented; in two patients the RBB had been detached and recemented once. Further, in one patient the RBB became detached more than once and in three patients (19%) more than five times.

### 2.2 Treatment Procedures and Equipment for Sensorimotor Measurements

**FIGURE 2:**
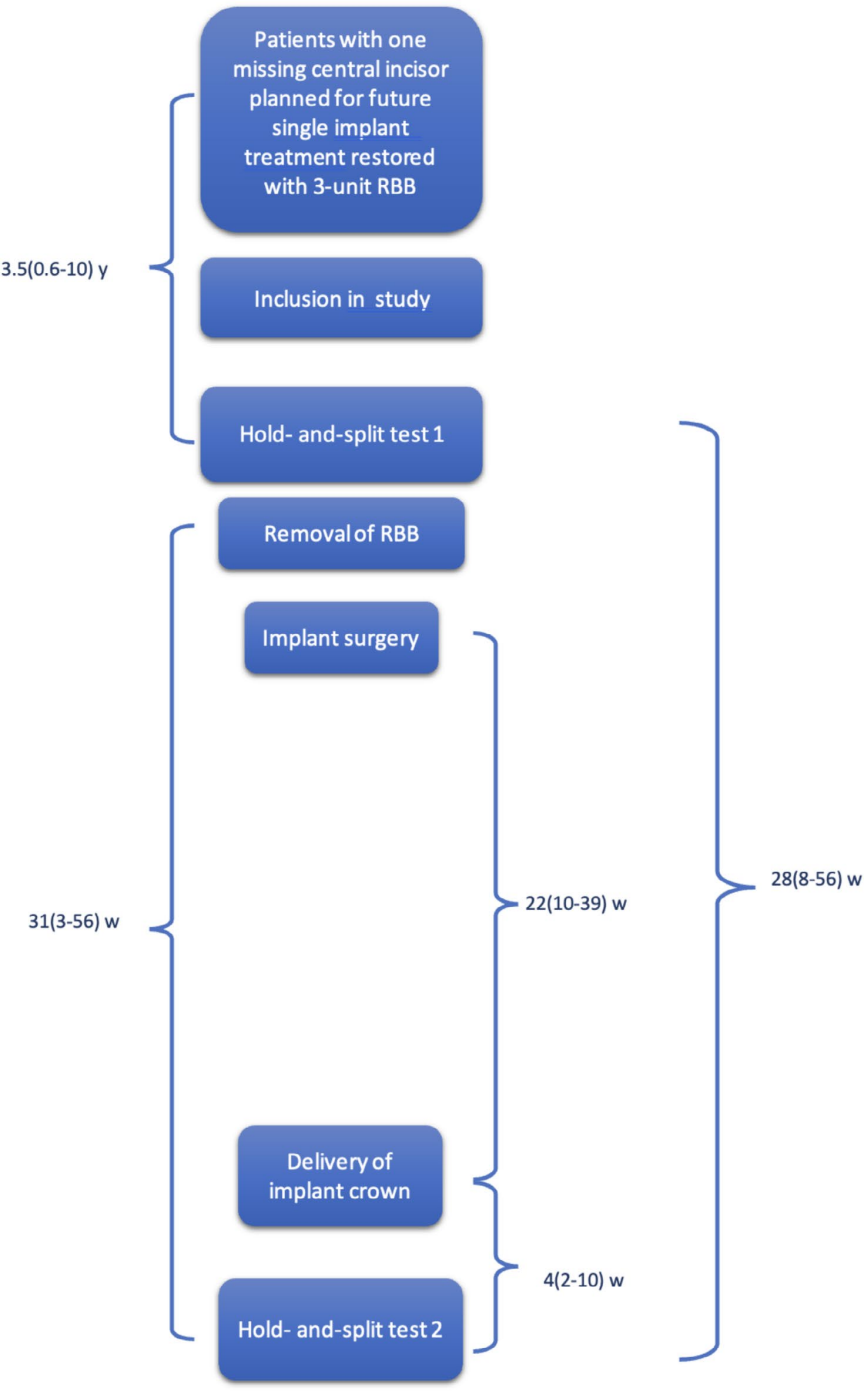
Flowchart illustrating the treatment and experimental procedures. Median time with ranges is reported (*n* = 16). y, years; w, weeks.

**FIGURE 3:**
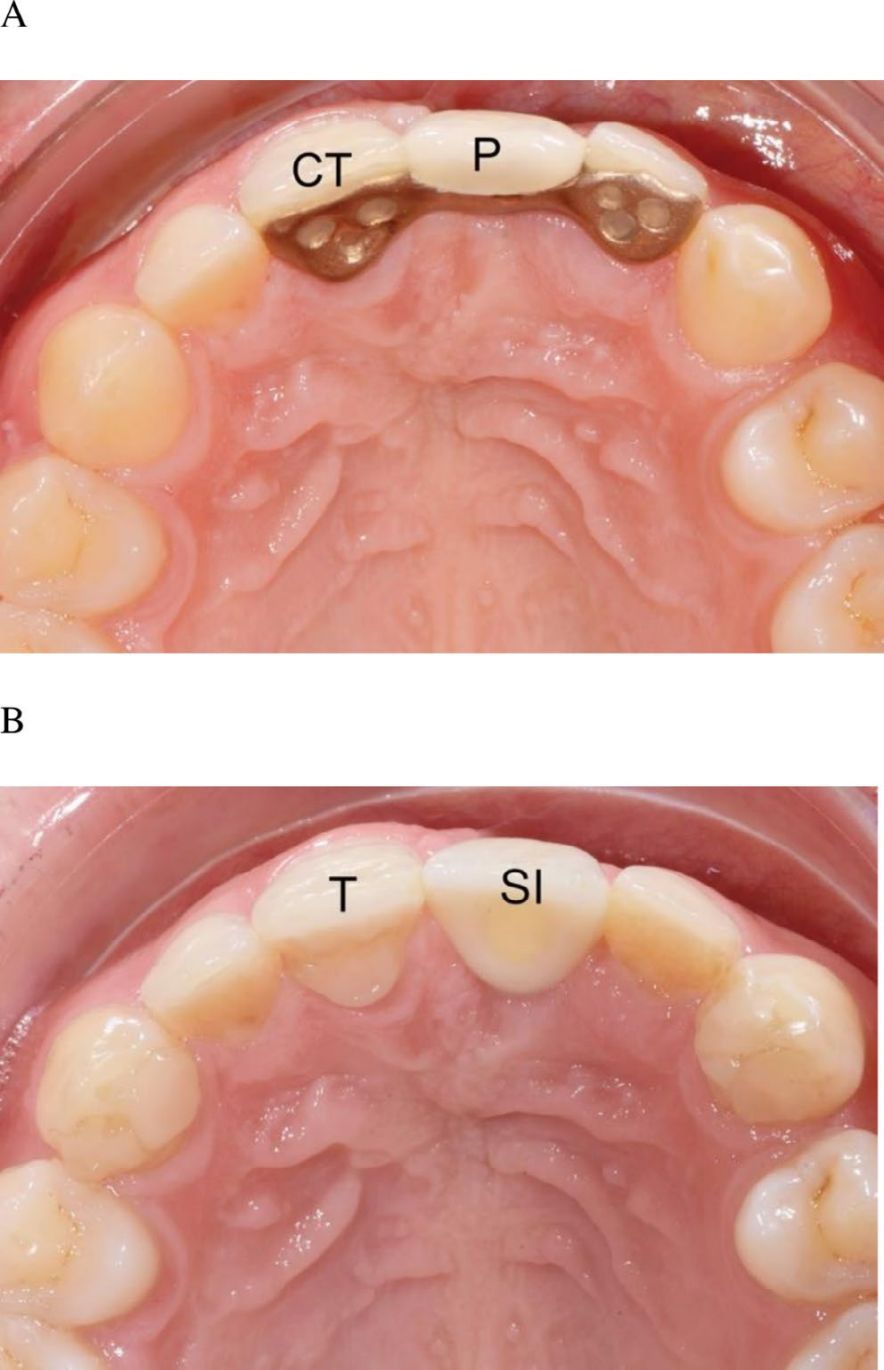
Pictures showing four different conditions tested with the hold‐and‐split task. (A) RBB in place, conditions CT (connected tooth) and P (pontic). (B) after implant placement, conditions T (free‐standing tooth) and SI (single implant).

The behavioral task to hold and split half a peanut was performed in a quiet room in a comfortable chair in front of a table. A custom‐built apparatus (Figure 1) was used (DC 200 Hz; Department of Integrative Medical Biology, Umeå University, Umeå, Sweden) to measure the hold‐and‐split forces (Al‐Manei et al. 2021). Four different conditions were tested at two different experimental sessions, before and after single implant treatment (Figure 2). Hence, the hold‐and‐split task was performed once on the pontic (P) of the RBB, once on the single implant (SI) and twice on the same central incisor; the first time connected to the RBB (CT) and the second time free‐standing adjacent to the single implant (T) (Figure 3). According to previous experiments with the same equipment, the participants were instructed to sit up straight and to hold the force transducer with one hand with the elbow resting on the table to ensure a horizontal relationship of the anterior teeth to the upper transducer's plate (Al‐Manei et al. 2021; Svensson and Trulsson 2011). After placing half a peanut (Estrella AB, Angered, Sweden) on the force transducer, the patient was asked to hold the nut between one upper central incisor and its antagonist with “as low force as possible” for about 3–4 s (hold phase) and then to split the peanut when asked to do so (split phase). The entire experimental setup and the procedure have been described in detail in previous studies (Trulsson and Johansson 1996a; Kumar et al. 2019, 2017, 2014; Al‐Manei et al. 2021; Svensson and Trulsson 2011; Almotairy, Kumar, Noirrit‐Esclassan, and Grigoriadis 2020). At least five practice trials, or until the participants were feeling comfortable with the procedure were performed before starting the registration of the hold‐and‐split forces. Every patient had 5 trials registered.

### 2.3 Data Analysis

**FIGURE 4:**
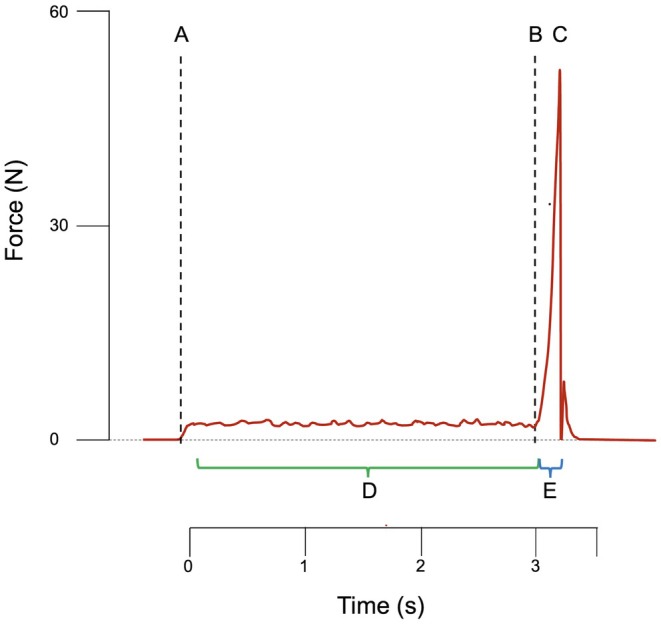
Illustration of force profile created by the forces applied at performance of the hold‐and‐split task. A, contact with peanut established. B, start of split phase. C, peak of split force. D, hold phase shown as fluctuating force line from 5 N/s after initial contact with peanut to 5 N/s before onset of split phase. E, split phase shown as rise in force until breaking of peanut occurs and force profile peaks and declines fast.

The force signals were sampled at 1000 Hz (low‐pass filtered 250 Hz) and analyzed with personal computer–based data acquisition and analysis software (WinSC/WinZoom; Department of Integrative Medical Biology, Umeå University, Umeå, Sweden). The force profile of each trial was divided into two distinguishable phases: the hold phase and the split phase in accordance with previous studies (Figure 4). The demarcations for the phases were detected by the computer system and manually checked for accuracy. The point for onset of the phases was chosen where the force rate exceeded 5 N/s, which is the minimum rate of increase that has been shown to be reliably detected during an individual trial (Svensson and Trulsson 2011) (Figure 4). The outcome variable variability of hold force was calculated by the standard deviation of hold force divided by the mean of the hold force during the individual trial. The split force was measured at the time when the peanut was split, which is seen on the force profile (Figure 4) as the peak after the rapid increase in force. The time required to reach the peak force of the split phase from the onset of the split phase was identified as the duration of the split.

### 2.4 Statistics

The estimation of effect size and required sample size was performed using G*Power software (version 3.1; Heinrich Heine University, Düsseldorf, Germany), based on data from comparable previous behavioral studies. Assuming a moderate effect size (Cohen's *d* = 0.6), with a Type I error rate (*α*) of 0.05 and a statistical power of 80% (*β* = 0.20), a minimum of 17 participants was required to detect the expected effect (Al‐Manei et al. 2021; Almotairy, Kumar, Welander, and Grigoriadis 2020). The outcome variables hold force, variability of hold force, split force, and duration of split between the two test conditions were presented as means ± standard deviation with confidence intervals (CI). To determine the normal distribution of the data, Shapiro–Wilk test, histograms, and normal Q‐Q plots were used. The normally distributed variable split force was analyzed with the paired student *t*‐test whereas the data presented with a skewed distribution (hold force, variability of hold force and duration of split) were analyzed with Wilcoxon signed rank test. Paired *T*‐tests were performed to analyze the difference between hold force at CT against T and P against SI for all subjects. *p*‐values < 0.05 were considered statistically significant, and for 95% CI not including 0 were considered statistically significant. The statistical analyses involved multiple paired, pre‐specified comparisons within a repeated‐measures design, in which each participant served as their own control. These comparisons were defined a priori based on biological and clinical relevance, rather than exploratory testing. Formal adjustment for multiple comparisons was therefore not applied, as such corrections may be overly conservative in small within‐subject experimental studies and may increase the risk of type‐II errors. Therefore, results were interpreted in conjunction with effect sizes, confidence intervals, and the consistency across outcome variables rather than isolated *p*‐values. Statistical analyses were performed using Stata/SE version 15 (StataCorp, College Station, TX, USA).

## 3 Results

All 16 participants completed both experimental sessions and were included in the statistical analyses. No missing data were recorded.

### 3.1 Hold Force

**FIGURE 5:**
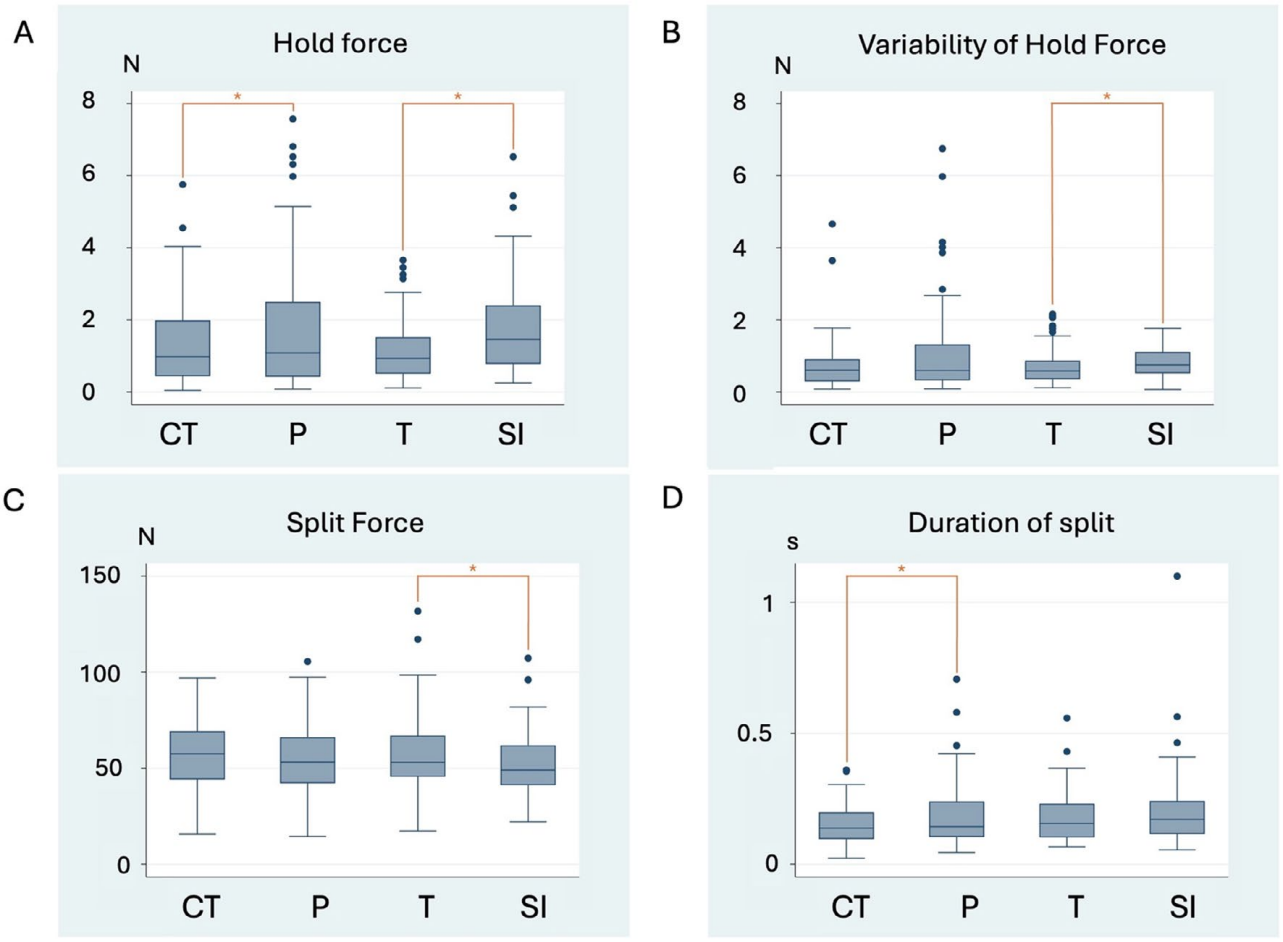
Box‐plot representation illustrating the differences in hold force, variability of hold force, split force, and duration of split at the four experimental conditions: Connected Tooth (CT), Pontic (P), Freestanding Tooth (T), and Single Implant (SI). The horizontal line within each box represents the median, the box boundaries indicate the interquartile range (IQR), and the whiskers extend to 1.5 × IQR. Dots denote outliers. Statistical significance was set at *p* < 0.05. (A) hold force. (B) variability of hold force. (C) split force. (D) duration of split. CT, connected tooth; P, pontic; T, free‐standing tooth; I, implant. N, Newton. s, time in seconds. *Statistically significant differences.

The mean and SD of hold force for condition CT were 1.32 ± 1.16 N, P 1.80 ± 1.87 N, T 1.14 ± 0.84 N, and SI 1.78 ± 1.28 N. The results of the statistical analysis showed significant differences between CT and P (*p* = 0.03), and T and SI (*p* = < 0.01). Contrary to our hypothesis, no significant differences between CT and T (*p* = 0.18) were found. Additionally, there were no significant differences between the P and SI (*p* = 0.36) (Figure 5, Table 1).

**TABLE 1:** Table presenting mean values with 95% confidence intervals (CI) for hold force, split force, duration, and variability of hold force under the different experimental conditions: Connected Tooth (CT), Freestanding Tooth (T), Pontic (P), and Single Implant (SI). Diff indicates the mean difference between compared groups.

Parameters	CT mean (95% CI)	T mean (95% CI)	Diff	*p*	P mean (95% CI)	SI mean (95% CI)	Diff	*p*
			CT–T				P–SI	
Hold force	1.14 (0.72–1.55)	1.14 (0.72–1.55)	0.00	0.988	1.74 (0.82–2.67)	1.71 (1.10–2.32)	0.04	0.947
Variability of hold force	0.71 (0.48–0.95)	0.63 (0.45–0.81)	0.09	0.470	0.91 (0.50–1.32)	0.78 (0.61–0.94)	0.14	0.470
Split force	50.86 (45.06–56.66)	47.41 (41.33–53.49)	3.45	0.390	46.50 (39.23–53.76)	43.73 (38.65–48.80)	2.77	0.413
Duration of split	0.15 (0.12–0.19)	0.17 (0.12–0.22)	−0.02	0.282	0.18 (0.11–0.25)	0.16 (0.13–0.19)	0.02	0.488

### 3.2 Variability of Hold Force

The mean and SD of variability of hold force for CT was 0.74 ± 0.71 N, P 1.07 ± 1.27 N, T 0.69 ± 0.47 N and SI 0.82 ± 0.43 N. Regarding the variability of hold force, no statistically significant differences between CT and P (*p* = 0.07), but a significant difference between T and SI (*p* = 0.02) was found. No significant differences between CT and T (*p* = 0.49) or between P and SI (*p* = 0.62) were found (Figure 5, Table 1).

### 3.3 Split Force

The mean and SD of split force for CT were 56.35 ± 16.99 N, P 55.22 ± 17.60 N, T 57.17 ± 18.87 N and SI 51.59 ± 16.11 N. The statistical analysis for the split force showed no significant differences between CT and P (*p* = 0.95), but a significant difference between T and SI (*p* = 0.05). Further, no significant differences were found between CT and T (*p* = 0.75) or between P and SI (*p* = 0.14) (Figure 5, Table 1).

### 3.4 Duration of Split

The mean and SD duration of split force for CT were 0.16 ± 0.08 s, P 0.19 ± 0.12 s, T 0.18 ± 0.09 s, and SI 0.20 ± 0.14 s. The results of the statistical analysis for the duration of split showed significant differences between CT and P (*p* = 0.03), but no significant difference between the T and SI (*p* = 0.29). Further, the results of the statistical analysis also showed no significant differences between CT and T (*p* = 0.06) or between P and SI (*p* = 0.62) (Figure 5, Table 1).

## 4 Discussion

Contradictory to our hypothesis, no significant difference in hold force, variability of hold force, split force, or duration of split was found when the natural tooth was connected in the RBB (CT) in comparison to being free‐standing adjacent to a single implant (T) nor was any significant difference found between the pontic (P) and single implants (SI). The lack of statistically significant differences between pontic and single implant should not be interpreted as superior oral fine motor control of the pontic. Instead, the two conditions showed comparable performance, with small mean differences and overlapping confidence intervals across outcome variables. Although the study may be underpowered to detect small effects, the findings suggest that any true difference is likely to be minor. This similarity is biologically possible, as both conditions lack direct periodontal mechanoreceptor input and rely on alternative sensory pathways. Significant differences in hold forces were found between CT and P and between T and SI. The study investigated the oral fine motor control in two different prosthetic constructions involving replacement of a single missing central incisor: a RBB and a SI. The oral fine motor control was tested at the four different conditions: CT, P, T, and SI. The findings suggest that teeth involved in 3‐unit anterior bridges seem to maintain the sensitivity in oral fine motor control with no significantly compromised force regulation during holding and splitting food.

Higher holding forces in the standardized hold‐and‐split task express impaired force control. The effect of impaired force control in patients with dental implants manifests as inability to efficiently bite (Grigoriadis et al. 2016), chew (Homsi et al. 2021, 2023) or adapt the jaw muscle activity to the hardness of the food (Grigoriadis et al. 2011; Grigoriadis and Trulsson 2018) compared to people with natural teeth. This has also been shown in patients with fixed full‐arch bimaxillary tooth supported bridges in comparison to natural dentate controls (Grigoriadis et al. 2016; Svensson and Trulsson 2011). It has been suggested that splinting teeth in rigid constructions weakens the sensory signals from the PMRs, which compromises the normal regulation of low contact (hold) and high biting (split) forces (Svensson and Trulsson 2011). The results of the current study with 3‐unit anterior bridges contradict previous results. The sensorimotor regulation (PMR stimulation) of teeth supporting the smaller anterior bridges seems to be affected in a different way than full‐arch bridges. One reason for this could be the mechanical advantages that apply for full‐arch bridges. The more extensive dimensioning of the material and the splinting of multiple teeth in a curved shape provides a more rigid construction. Further, the stress applied to incisors in full‐arch bridges is distributed to and shared with the more stable premolar and molar areas along the bridges (Weinberg 1957a, 1957b; Nyman and Lang 1994). The literature is inconsistent as to whether the tactile sensibility differs between natural single teeth and single implants (Enkling et al. 2012, 2007; Hammerle et al. 1995). Instead of relying on PMRs, the tactile sensibility of a single implant lacking periodontal membrane acts through osseoperception, where receptors within the mucosa, the alveolar bone, the chewing muscles, and the temporomandibular joint act as sensors (Jacobs and van Steenberghe 1991, 2006; Song et al. 2022). However, in agreement with previous findings (Svensson and Trulsson 2011; Trulsson and Gunne 1998) the significantly larger hold forces found at SI than T in the present study show that the PMRs provide a more sensitive functional response than osseoperception does. The significantly larger hold forces found on P than CT in the bridges were comparable to the relation between SI and T. This indicates an impaired force control on P compared to CT in the same manner as between SI and T, and thereby a resemblance in oral fine motor control of the two treatment modalities, anterior single implants and 3‐unit bridges.

Anterior teeth are provided with a vast number of PMRs and are sensitive to load in all directions (mesial, distal, lingual, facial, and up and down), while the distal teeth (premolars and molars) are presented with fewer PMRs and a directional sensitivity in only three of the six directions (lingual, distal, and downwards) (Trulsson 2006). The role of the incisors is to manipulate and cut food in the beginning of the mastication process. The sensitivity to load in all directions is thereby an important property for transferring information about the loads. The molars are not in need of the same sensitivity since their main function during mastication is to grind and more forcefully chew the food morsels after the split has been performed (Johnsen and Trulsson 2003). The mean hold forces of the free‐standing tooth measured in the present study was 1.14 ± 0.84 N. These forces are in the same order of magnitude as where the PMR's at the incisors show their highest sensitivity and activity (around 1 N) (Trulsson 2005, 2006; Trulsson and Johansson 1996a). Furthermore, about 50% of human PMR's can respond to input from more than one tooth. Through so‐called receptive fields, adjacent teeth can also be stimulated upon loading of one specific tooth (Trulsson 1993). The stimulated tooth shows the highest sensitivity upon loading, while the adjacent teeth provide additional important information about loading and its directions. This transferal of force to adjacent teeth is presumed to be caused by physical coupling (interdental contacts and transseptal collagen fibers) rather than branching of single periodontal mechanoreceptors afferents (Trulsson 1993). While the loading of a stimulated tooth has been shown to be evenly distributed over four directions, namely lingually, buccally, mesially and distally, the loading of the adjacent tooth primarily showed the strongest response in the mesial and distal directions, often towards the most sensitive adjacent tooth (Trulsson 1993). Trulsson and coworkers have proposed that if the information of one tooth is lost, information about tooth loading will still be available from the neighboring teeth (Trulsson 1993). In the present study, the high sensitivity of the anterior teeth and the presence of receptive fields could also explain the failure to detect differences between the free‐standing tooth and the tooth connected in a bridge.

The strength of the present study was that the study design allowed for the patients to serve as their own controls while being tested with a previously used standardized method (Kumar et al. 2019; Al‐Manei et al. 2021; Svensson and Trulsson 2011; Almotairy, Kumar, Noirrit‐Esclassan, and Grigoriadis 2020; Trulsson and Gunne 1998). Limitations needed to be mentioned are that the time with a RBB and in place (0.6–9 years) and the number of times that the bridge had become loose (0–> 5 times) differed between patients before the first hold‐and‐split test. Also, the time with the single implant crown in function before the second test differed (2–9 weeks). These circumstances might have made some patients more secure/familiar with the RBB/single implant crown than others, which could affect the performance in the behavioral tasks. Due to the demographic distribution in the study being skewed toward younger patients (mean age 24 ± 9 years) and the primary cause of tooth loss being trauma, the external validity of the findings is limited. Consequently, the results may not be generalizable to older patients or patients with teeth with reduced periodontal support, in whom oral fine motor control may be more restricted (Johansson et al. 2006). The relatively small sample size limits the ability to detect small effects, and the possibility of a type‐II error cannot be fully excluded. Therefore, non‐significant findings should be interpreted with caution, particularly when expected differences between conditions are subtle. Further, the hardness of the peanuts can differ within a bag of peanuts and also between bags. Additionally, this kind of tests also depends on the patients' state of mind. Efforts were made to make the environment as calm and relaxed as possible and not to differ between the patients, but personal factors within the patients can still affect their behavior during the testing. Although the number of patients in the study equals or even exceeds the patient material in previous studies using the same test method (Kumar et al. 2019; Al‐Manei et al. 2021; Svensson and Trulsson 2009; Johansson et al. 2006; Trulsson and Gunne 1998) it is still a fairly small material.

Within the limitations of this study, the sensorimotor regulation properties of anterior teeth supporting a 3‐unit RBB do not seem to be considerably compromised compared to the free‐standing teeth adjacent to dental implants, despite the “splinting” of the teeth. The oral fine motor control of a pontic in a 3‐unit RBB seems to be comparable to a single implant. Clinically, in terms of affecting oral fine motor control in patients with single anterior tooth loss, an RBB seems to be comparable to treatment with a single implant.

## 5 Conclusions

Based on the findings of this clinical experimental study, the following conclusions were drawn:

1. Contradicting our hypothesis, no significant differences in oral fine motor control were found between the tooth splinted in a 3‐unit anterior RBB (CT) and a freestanding tooth (T) adjacent to a single implant. These results indicate that both treatment modalities may provide comparable outcomes in terms of oral fine motor control.
2. The oral fine motor control on a single implant (SI) was compromised in comparison to T and comparable to the relation of sensitivity of a pontic (P) to a CT of a 3‐unit RBB.

## Author Contributions

**Nicole Winitsky:** conceptualization, investigation, writing – original draft, validation, visualization, writing – review and editing, formal analysis, project administration. **Jan‐Ivan Smedberg:** funding acquisition, writing – review and editing, project administration, supervision. **Anastasios Grigoriadis:** conceptualization, methodology, validation, writing – review and editing, data curation, formal analysis, supervision. **Aron Naimi‐Akbar:** conceptualization, methodology, writing – review and editing, formal analysis, supervision, data curation.

## Funding

This work was supported by grants from Folktandvården Stockholm AB, the Stockholm County Council, and Karolinska Institutet (SOF: Styrgruppen för Odontologisk Forskning).

## Conflicts of Interest

The authors declare the following financial interests/personal relationships which may be considered as potential competing interests. Nicole Winitsky reports a relationship with Nobel Biocare Services A.G. that includes: consulting or advisory and speaking and lecture fees. Nicole Winitsky reports a relationship with Neoss Inc. that includes: speaking and lecture fees. Nicole Winitsky reports a relationship with Perrigo Co Plc that includes: consulting or advisory. The other authors declare no conflicts of interest.

## Supporting information


**Data S1:** STROBE Statement—Checklist of items that should be included in reports of cohort studies.

## Data Availability

The data that support the findings of this study are available on request from the corresponding author. The data are not publicly available due to privacy or ethical restrictions.
