# Supplementary material for: Oral Fine Motor Control in Patients With Anterior Maxillary Single Implants, Freestanding Teeth and Teeth Connected in 3‐Unit Bridges: An Experimental In Vivo Study
Source: Clin Oral Implants Res. 2026 May 8;37(8):922–30. doi: 10.1111/clr.70136 (PMC13446234; doi:10.1111/clr.70136)
Supplement: Supplementary file 1 [file CLR-37-922-s001.docx]

# STROBE Statement—Checklist of items that should be included in reports of cohort studies

| Item No. | Recommendation (Official STROBE wording – Cohort) | Page No. | Location in manuscript |
| --- | --- | --- | --- |
| 1a | Indicate the study’s design with a commonly used term in the title or the abstract | 1, 6 | Title (p1); Materials and Methods first paragraph (p6). |
| 1b | Provide in the abstract an informative and balanced summary of what was done and what was found | 4 | Abstract page. |
| 2 | Explain the scientific background and rationale for the investigation being reported | 5–6 | Introduction section. |
| 3 | State specific objectives, including any prespecified hypotheses | 6 | Final paragraph of Introduction. |
| 4 | Present key elements of study design early in the paper | 6 | Materials and Methods first paragraph. |
| 5 | Describe the setting, locations, and relevant dates, including periods of recruitment, exposure, follow-up, and data collection | 7 | Materials and Methods and Patients sections. |
| 6a | Give the eligibility criteria, and the sources and methods of selection of participants. Describe methods of follow-up | 6–7 | Materials and Methods; Patients. |
| 6b | For matched studies, give matching criteria and number of exposed and unexposed | N/A | Not a matched study. |
| 7 | Clearly define all outcomes, exposures, predictors, potential confounders, and effect modifiers | 7–9 | Treatment procedures; Data analysis; Statistics. |
| 8 | For each variable of interest, give sources of data and details of methods of assessment | 7–8 | Treatment procedures and Data analysis sections. |
| 9 | Describe any efforts to address potential sources of bias | 13–14 | Discussion limitations paragraph. |
| 10 | Explain how the study size was arrived at | 8–9 | Statistics section (G*Power calculation). |
| 11 | Explain how quantitative variables were handled in the analyses | 8–9 | Statistics section. |
| 12a | Describe all statistical methods, including those used to control for confounding | 8–9 | Statistics section. |
| 12b | Describe any methods used to examine subgroups and interactions | N/A | No subgroup analyses. |
| 12c | Explain how missing data were addressed | 9 | Results first paragraph. |
| 12d | If applicable, explain how loss to follow-up was addressed | 9 | Results first paragraph (no loss to follow-up). |
| 12e | Describe any sensitivity analyses | N/A | No sensitivity analyses performed. |
| 13a | Report numbers of individuals at each stage of study | 7, 9, 20 | Patients (p7); Results (p9) |
| 13b | Give reasons for non-participation at each stage | N/A | No non-participation reported. |
| 13c | Consider use of a flow diagram | 20 | Figure 2 (Flowchart). |
| 14a | Give characteristics of study participants and information on exposures and potential confounders | 7 | Patients section. |
| 14b | Indicate number of participants with missing data for each variable of interest | 9 | Results first paragraph (no missing data). |
| 14c | Summarise follow-up time | 20 | Figure2 (Flowchart). |
| 15 | Report numbers of outcome events or summary measures over time | 9–10, 23–24 | Results section; Figure 5 (p23); Table 1 (p24). |
| 16a | Give unadjusted estimates and, if applicable, confounder-adjusted estimates and their precision | 9–10, 24 | Results; Table 1 with 95% CI. |
| 16b | Report category boundaries when continuous variables were categorized | N/A | Continuous variables not categorized. |
| 16c | If relevant, consider translating estimates into absolute risk for a meaningful time period | N/A | Not applicable to physiological outcomes. |
| 17 | Report other analyses done—eg analyses of subgroups and interactions, and sensitivity analyses | N/A | No additional analyses. |
| 18 | Summarise key results with reference to study objectives | 10–11 | Discussion first paragraph. |
| 19 | Discuss limitations of the study, taking into account sources of potential bias or imprecision | 13–14 | Discussion limitations section. |
| 20 | Give a cautious overall interpretation of results considering objectives, limitations, multiplicity of analyses, results from similar studies, and other relevant evidence | 11–14 | Discussion section. |
| 21 | Discuss the generalisability (external validity) of the study results | 13–14 | Discussion limitations paragraph. |
| 22 | Give the source of funding and the role of the funders | 2 | 'This study received no external funding.' |
